# Severe degeneration of a sub-coronary pulmonary autograft in a young adult

**DOI:** 10.21542/gcsp.2021.14

**Published:** 2021-06-30

**Authors:** Najma Latif, Ahmed Mahgoub, Mohamed Nagy, Padmini Sarathchandra, Magdi H. Yacoub

**Affiliations:** 1 Magdi Yacoub Institute, Heart Science Centre, Harefield Hospital, Harefield, Middx UB9 6JH, UK; 2 National Heart and Lung Institute, Imperial College London, ICTEM Building, London, UK; 3 Aswan Heart Centre, Aswan, Egypt

## Abstract

**Background.** The pulmonary autograft is currently the best valve substitute in terms of longevity and performance. However, there is no agreement about the optimal method of insertion (sub-coronary position or freestanding root).

**Objectives.** We sought to examine the clinical status, detailed imaging and morphometric changes in an explanted pulmonary autograft 22 years after sub-coronary implantation.

**Methods.** A 30-year-old female underwent pulmonary autograft replacement of a severely stenotic valve at the age of 7 years, after presenting to us with signs of moderate to severe heart failure. She underwent clinical examination, detailed imaging including echocardiographic and CT examination with computerised image analysis. The explanted valve was examined by morphometry.

**Results.** Clinical examination showed signs of heart failure (NYHA III). Trans-thoracic and trans-oesophageal 2D echo showed severe malfunction of both the aortic and pulmonary valves associated with dilatation and hypertrophy of both the right and left ventricles. Surgical correction was performed by replacing both the pulmonary and aortic valves with Medtronic 27mm Freestyle valves. The pulmonary autograft showed degeneration of the trilamellar layering of the leaflets, loss and disorganisation of GAGs, increased collagen with fibrotic overgrowth, and markers of fibrosis, inflammation, and calcification. Post-operative imaging showed good correction of the haemodynamic lesions.

**Conclusion.** The pulmonary autograft implanted into the sub-coronary position presented with adverse remodelling, which was detrimental to the functionality and longevity of the valve.

**Authorship.** NL, AM, MN all contributed equally to this paper.

## Introduction

Pulmonary autograft replacement of the aortic valve (Ross operation) is currently the most durable form of aortic valve replacement^1–6^. This is closely linked to the capacity of the valve substitute to remain viable and adaptive to local and systemic conditions, both in the short and long term^1,7,8^. The original operation was configured in the sub-coronary position by Donald Ross^9^ and was modified to a freestanding root replacement (previously described for homografts)^10^.

However, varying degrees of degenerative changes, resulting in malfunction of the pulmonary autograft, have been reported. The exact incidence and, importantly, the factors contributing to the degenerative changes, have not been adequately defined. We describe the evolution of long-term severe degeneration of a pulmonary autograft, inserted in the sub-coronary position and discuss the possible role of the method of insertion on this serious complication. In addition, we compare these changes to those seen following the root replacement technique^7^.

## Methods

### Sample preparation

Paraffin-embedded sections of valve leaflets were examined with histochemical and immunohistochemical staining as previously described^7^.

## Results

### Clinical course and imaging

A 30-year-old female had a sub-coronary root replacement at the age of 7. This patient presented with progressive dyspnea on exertion from the age of 16 (NYHA III). On examination she was found to have mild central cyanosis, congested neck veins, pan-systolic murmur over the pericardium, and marked hepatosplenomegaly.

**Figure 1.**
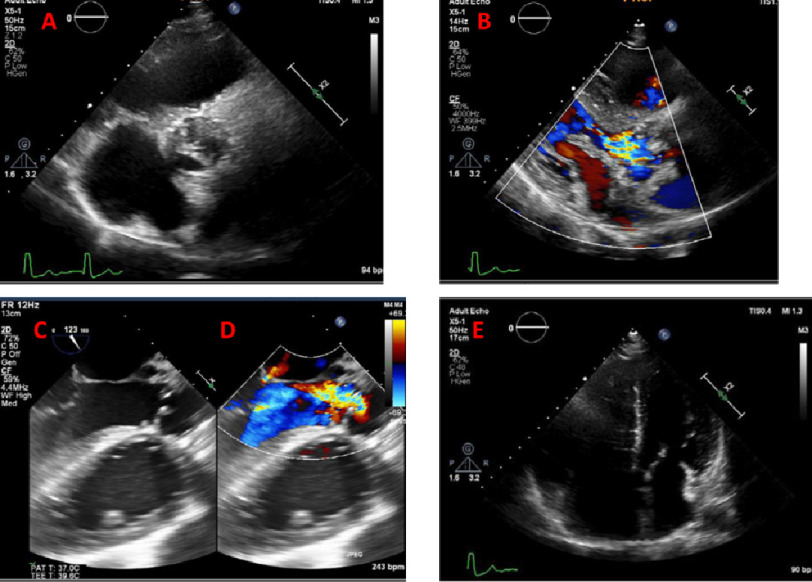
Trans-thoracic echo, short axis view at level of aorta showing calcified leaflets with restricted closure and mal-coaptation (A). Trans-thoracic echo, parasternal long axis view showing color Doppler of the aortic valve with severe aortic regurgitation (B). Transesophageal echo, 120-degree view showing the aortic valve with restricted calcific leaflets in 2D (C) and color Doppler showing severe aortic regurgitation (D). Trans-thoracic echo, apical four chamber view showing dilated hypertrophied RV due to pressure overload (E).

**Figure 2.**
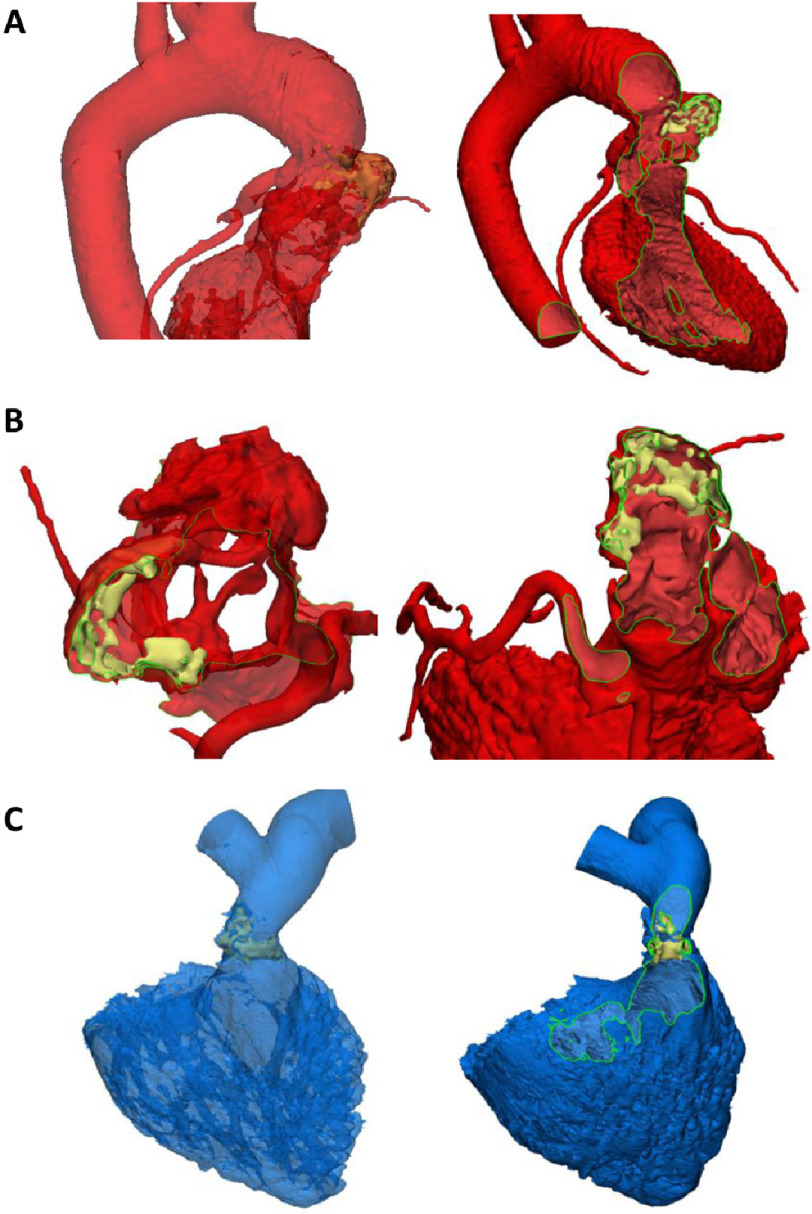
MSCT derived 3D models of the aortic root showing severe distortion of the aortic root with aneurysmal dilatation above the right coronary sinus (A) as well as significant stenosis of the origin of the right coronary (A). Severe calcification involving parts of the leaflets and the inside of the aneurysm (B). 3D models of the RV and outflow showing severe calcification and stenosis of the homograft conduit inserted 23 years previously (C).

Echocardiography findings (Figure 1) showed severe aortic regurgitation and moderate aortic stenosis (Figure 1A). Multislice computed tomography (MSCT) angiography demonstrated LVOT pseudoaneurysm, heavily calcific aneurysmal dilated right coronary cusp, significant osteal stenosis of right coronary artery (Figure 2A), heavily calcific pulmonary valve with thickened leaflets, valvular and supravalvular pulmonary stenosis (Figure 2B), dilated aortic root and ascending aorta (Figure 2A), dilated right side with hypertrophied RV (Figure 2C).

**Figure 3.**
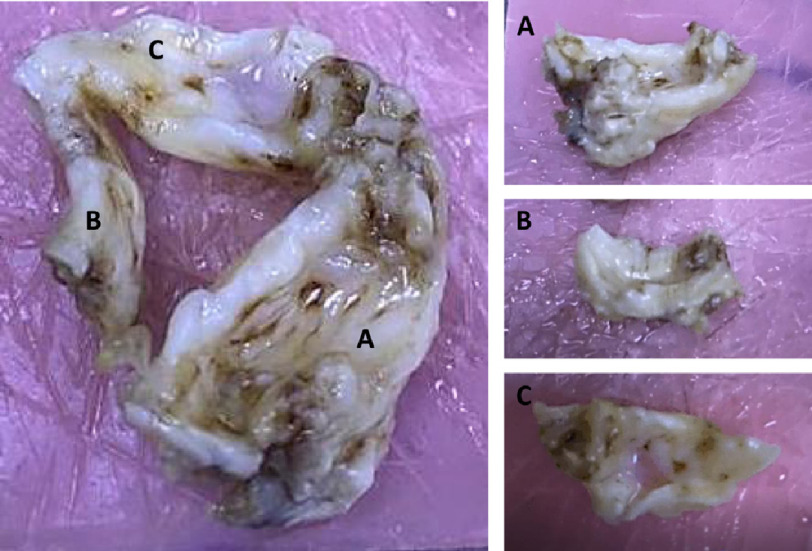
Macroscopic view of all the leaflets.

The ICU course necessitated prolonged mechanical ventilation due to a lung collapse, which gradually improved. Additionally, the patient suffered from atrial fibrillation and atrial flutter attacks, which were successfully cardioverted back to a normal sinus rhythm. The sub-coronary root replacement was explanted 23 years later (Figure 3) after a redo sternotomy, and both pulmonary and aortic valves were replaced by 27 mm Freestyle valves.

**Figure 4.**
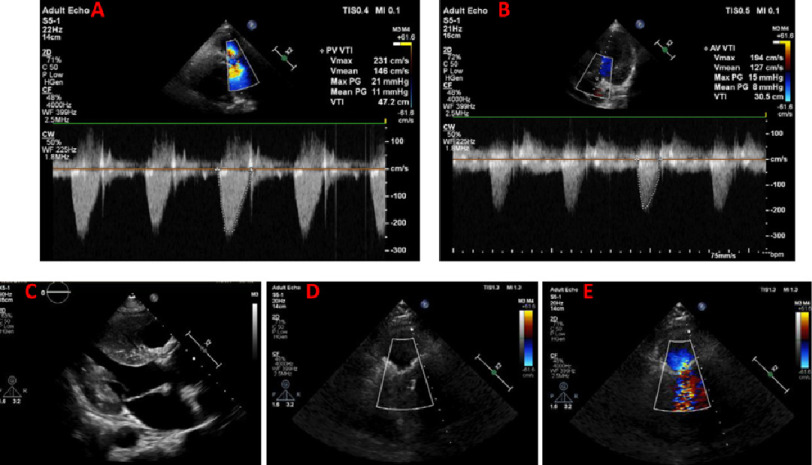
Continuous wave Doppler over neo-pulmonary valve showing no significant gradients across valve 1-month post-surgery (A). Continuous wave Doppler over neo-aortic valve showing no significant gradients across valve 1-month post-surgery (B). 2D echocardiogram of the neo-aortic valve (Freestyle prosthesis) with full leaflet motion (C). RV outflow view showing the neo-pulmonary valve with full leaflet motion in 2D (D) and color Doppler showing laminar flow across the valve (E).

At follow up, the patient was asymptomatic with normal sinus rhythm, normal LV function and dimensions and no significant gradients across either valve (Figure 4).

### Morphometric examination of the explanted pulmonary autograft

**Figure 5.**
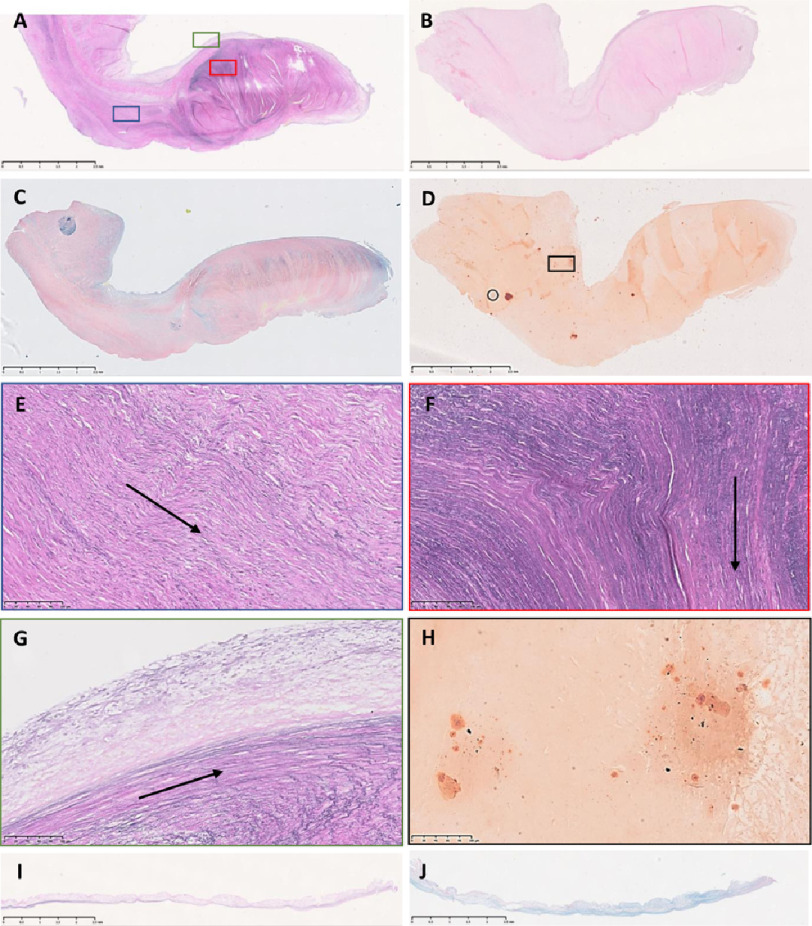
Sections from leaflet A stained with elastin van Gieson (A), haemotoxylin and eosin (B), Alcian blue/Sirius red (C) and alizarin red (D). Colour-coded magnified views of panels in A (E–G) and in panel D (H). A normal pulmonary valve stained with elastin van Gieson (I) and Alcian blue/Sirius red (J). The right side of each image is the co-apting edge and the left side is the hinge side. The top is the fibrosal side and the bottom the ventricularis. Scale bar represents 2.5 mm in top 4 panels, bottom 2 panels and 100 μm in other panels.

**Figure 6.**
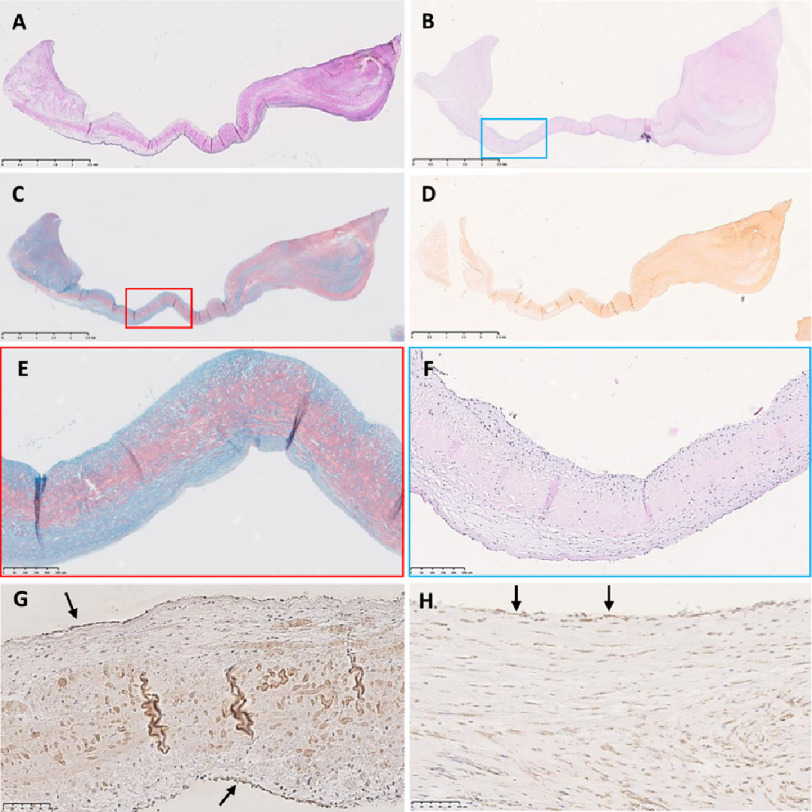
Sections from leaflet B stained with elastin van Gieson (A), haemotoxylin and eosin (B), Alcian blue/Sirius red (C), alizarin red (D), colour coded magnified views of panels in C (E) and in panel B (F). Positive endothelial staining for α-SMA (Figure 6G) and Runx2 (Figure 6H). The right side of each image is the co-apting edge and the left side is the hinge side. The top is the fibrosal side and the bottom the ventricularis. Scale bar represents 2.5 mm in top 4 panels, 250 μm in the next row and 100 μm in the bottom 2 panels.

**Figure 7.**
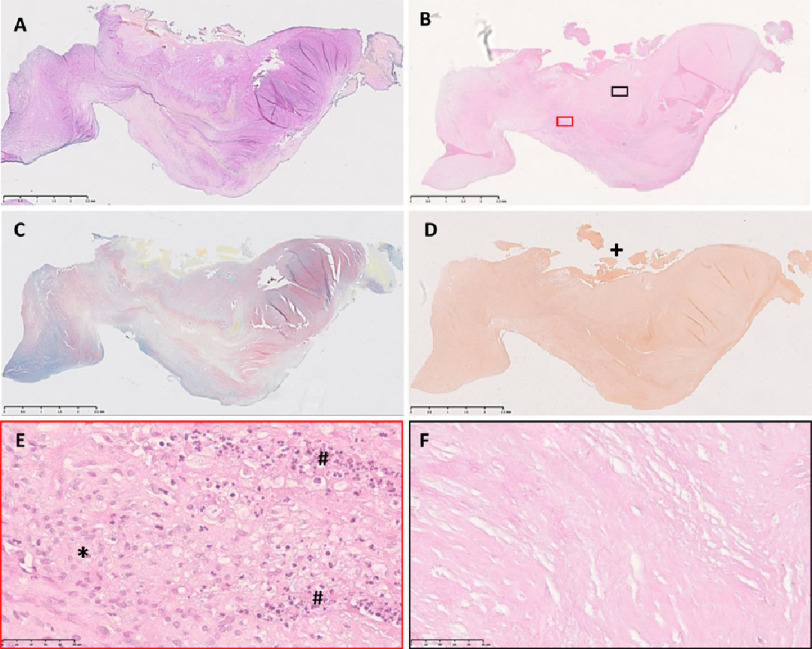
Sections from leaflet C stained with elastin van Gieson (A), Alcian blue/Sirius red (C), haemotoxylin and eosin (B) and alizarin red (D), colour coded magnified views of panels in B (E,F). The right side of each image is the co-apting edge and the left side is the hinge side. The top is the fibrosal side and the bottom the ventricularis. Scale bar represents 2.5 mm in top 4 panels, 50 μm in panel E and F.

All 3 leaflets of the pulmonary explant showed thickening, stiffening and an irregular, clumpy surface (Figure 3). Leaflets B and C showed only a small region with any semblance of normal valve tissue (Figures 3B, 3C). The maximal thickening extended to 3.86 mm, 4.18 mm (thickened section not shown) and 4.62 mm in leaflet A, B, and C respectively (Figures 5–7).

### EVG

The amount and distribution of elastin was increased (Figure 5). This was mostly in the radial direction with many separate parallel layers of elastin at both ventricular (Figure 5E) and fibrosal sides (Figure 5G) of the leaflets however in thickened regions, fibres of elastin were discernible in anteroposterior direction (Figure 5F) and disorganised (Figure 5G). The elastin in the normal pulmonary leaflet was restricted predominantly to the ventricularis (Figure 5I). Collagen was increased in all layers of the Ross leaflets (Figure 5C) compared to the fibrosal pattern in the normal pulmonary leaflet (Figure 5J).

### ABSR

The central glycosaminoglycan (GAG) layer was disrupted in all leaflets with a random distribution of GAGs and abundant collagen (Figures 5C, 6C and 7C). GAGs were increased in 2 leaflets (Figures 6C and 7C) and in one of these leaflets, these were expressed abnormally at the edges of the leaflet (Figures 6C, 6E) despite having a normal thickness. Collagen was distributed throughout leaflet A (Figure 5C), present in the central and thickened region of leaflet B (Figures 6C, 6E) and disorganised throughout leaflet C (Figure 7C).

### Immunostaining

**Figure 8.**
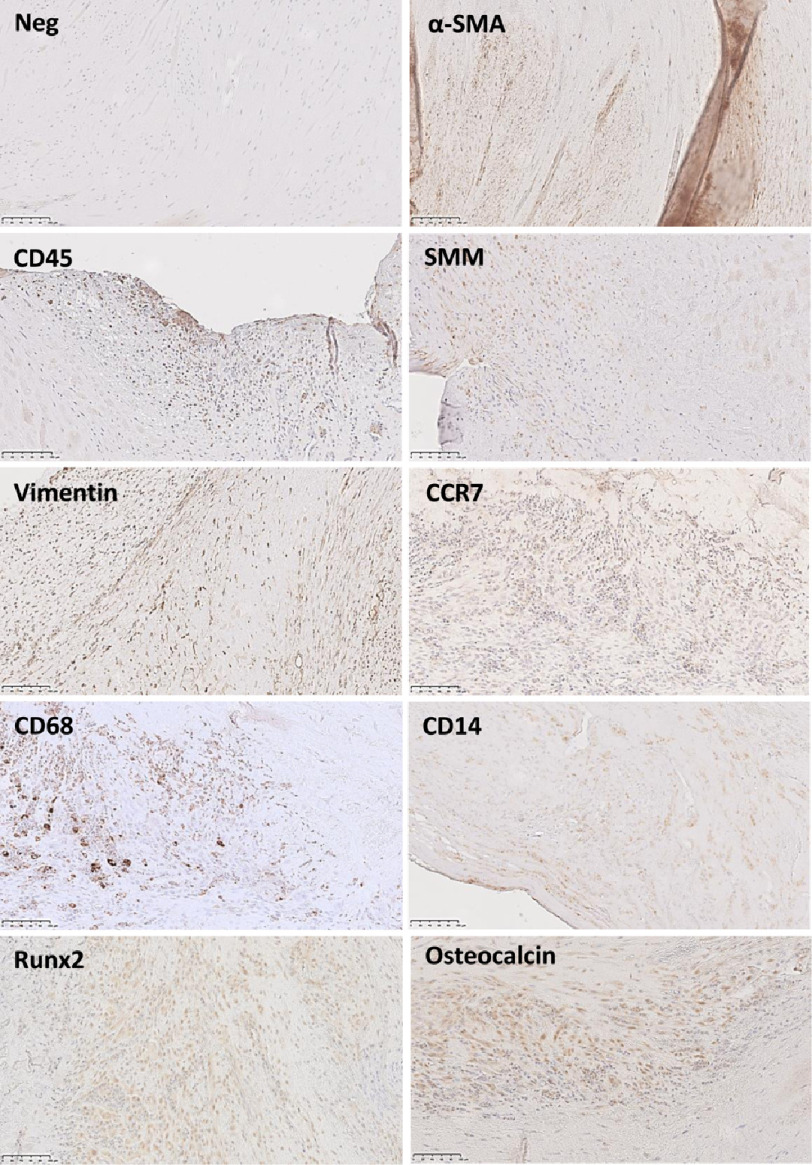
Panels show immunostaining of specific markers. Scale bar represents 100 μm.

Cellularity was reduced markedly in the central regions of all the thickened leaflets and absent in some regions (Figures 7B, 7F). However, the fibrotic overgrowth regions on both sides of the leaflets contained numerous cells (Figure 7E). The only region of normal thickness on leaflet B showed a normal distribution of valve interstitial cells, however these cells showed an abnormal phenotype with a similar pattern of expression of calcification markers, Runx2 and osteocalcin (Figure 8).

On higher magnification, the native valve interstitial cells, with larger, elongated nuclei, could be discerned from the smaller, infiltrating polymorphonuclear cells (Figure 7E) and these latter cells were abundant in all leaflets. Many myofibroblasts (staining for α-smooth muscle actin) and leucocytes (CD45) were present in the cellular regions (Figure 8) with a small population of smooth muscle cells (Figure 8, SMM). No T cells were identified, however macrophages (CD68), and monocytes (CD14) were identified, with macrophages being predominantly of the M1 phenotype (CCR7). Activation of valve interstitial cells was evident by expression of α-SMA and Runx2 (Figure 8) as well as in endothelial cells (Figures 6G, 6H), even in macroscopically-normal tissue.

### Calcification

Despite being markedly thicker and stiffer than normal aortic valves (Figure 3), there was minimal alizarin red staining in the leaflets (Figures 5D, 5H and 7D). The fibrosal sides of leaflets B and C showed some darker staining for alizarin red (Figure 7D), while leaflet A showed a spotted pattern on both sides of the leaflet (Figure 5H). Markers for calcification, Runx2 and osteocalcin were, however abundant in all leaflets in valve interstitial cells (Figure 8) and also valve endothelial cells (Figure 6G).

### Discussion and Conclusion

This manuscript documents the long-term adverse effects of the use of the sub-coronary position for the Ross operation, which can potentially introduce distortion both in the aortic root and components of the valve culminating in late failure. In contrast, the use of the freestanding aortic root, which was introduced for homografts very early on^10^, guarantees an optimal relationship between the component parts of the valve.

**Figure 9.**
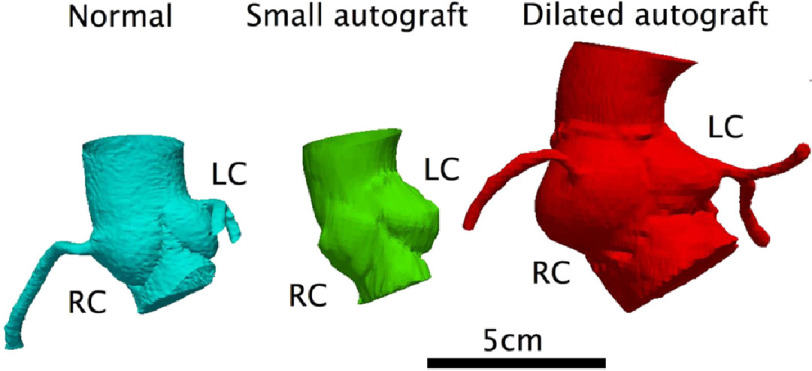
CT-generated 3D models of the freestanding aortic root showing variable degrees of dilatation.

**Figure 10.**
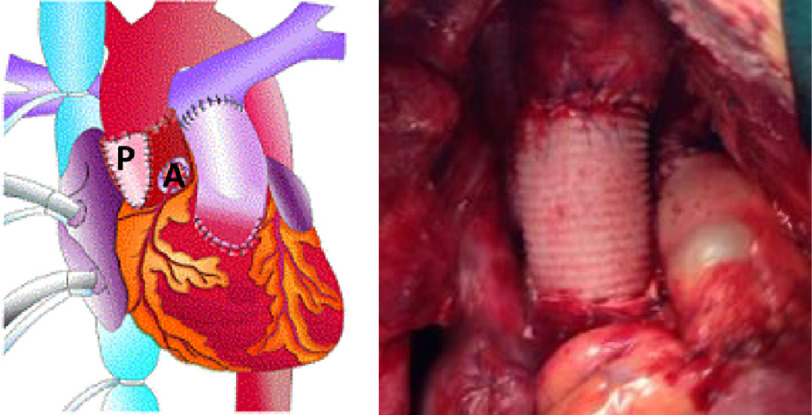
The use of autologous tissue (autologous aortic wall and pericardium) to support the freestanding root (the loose jacket technique) as used in our centre.

Interestingly, long term follow-up of homografts showed that the use of a freestanding root was associated with better survival^11^. There is a continuing concern about the use of the freestanding root for pulmonary autografts because of the reports of dilatation of the root, resulting in malfunction of the valve^12–16^. This has stimulated the use of the sub-coronary implantation as the autograft is supported by the native valve preventing dilatation^17^. However as is illustrated from the current patient, accurate positioning of the pulmonary autograft in the sub-coronary position requires both skill and judgement, particularly when there is a mismatch between the size and shape of the root as compared to the autograft (Figure 9). Regarding the dilatation following the free root replacement, this is quite variable and occurs in only a small fraction of patients (Figure 9). To safeguard against this complication the technique of loose jacket is being used routinely in our centre (Figure 10).

To date, there has been no randomised trials comparing the two techniques, however a systematic review performed by Berdajs and Von Segesser showed better results with the root replacement, both in terms of longevity and incidence of reoperation^18^. It is hoped that the data presented in this manuscript can be of some help in decision making in the choice of technique in the Ross operation.

### Lessons Learnt

1. The Ross operation continues to be the best aortic valve substitute, particularly in children.
2. Sub-coronary implantation can result in both distortion of the root and degeneration of the component parts.
3. The use of root replacement guarantees appropriate relationship between the component parts of the aortic root^10^ and valve, and has been shown to have a survival advantage following homograft valve replacement^11^.
4. Late dilatation of the aortic root when inserted as freestanding can be prevented (at least in theory) with the use of an autologous loose jacket (19).
